# Development of a Novel Perfusable Solution for *ex vivo* Preservation: Towards Photosynthetic Oxygenation for Organ Transplantation

**DOI:** 10.3389/fbioe.2021.796157

**Published:** 2021-12-15

**Authors:** Valentina Veloso-Giménez, Rosalba Escamilla, David Necuñir, Rocío Corrales-Orovio, Sergio Riveros, Carlo Marino, Carolina Ehrenfeld, Christian Dani Guzmán, Mauricio P. Boric, Rolando Rebolledo, José Tomás Egaña

**Affiliations:** ^1^ Schools of Engineering, Medicine and Biological Sciences, Institute for Biological and Medical Engineering, Pontificia Universidad Católica de Chile, Santiago, Chile; ^2^ Department of Physiology, Faculty of Biological Sciences, Pontificia Universidad Católica de Chile, Santiago, Chile; ^3^ Division of Hand, Plastic and Aesthetic Surgery, LMU Munich, University Hospital, Munich, Germany; ^4^ Department of Digestive Surgery, Faculty of Medicine, Pontificia Universidad Católica de Chile, Santiago, Chile; ^5^ Sky-Walkers SpA, Litueche, Chile; ^6^ Hepatobiliary and Pancreatic Surgery Unit, Surgery Service, Hospital Dr. Sótero del Río, Santiago, Chile

**Keywords:** organ preservation, organ perfusion, ischemia, hypoxia, photosynthetic microorganisms, *Chlamydomonas reinhardtii*, photosynthesis

## Abstract

Oxygen is the key molecule for aerobic metabolism, but no animal cells can produce it, creating an extreme dependency on external supply. In contrast, microalgae are photosynthetic microorganisms, therefore, they are able to produce oxygen as plant cells do. As hypoxia is one of the main issues in organ transplantation, especially during preservation, the main goal of this work was to develop the first generation of perfusable photosynthetic solutions, exploring its feasibility for *ex vivo* organ preservation. Here, the microalgae *Chlamydomonas reinhardtii* was incorporated in a standard preservation solution, and key aspects such as alterations in cell size, oxygen production and survival were studied. Osmolarity and rheological features of the photosynthetic solution were comparable to human blood. In terms of functionality, the photosynthetic solution proved to be not harmful and to provide sufficient oxygen to support the metabolic requirement of zebrafish larvae and rat kidney slices. Thereafter, isolated porcine kidneys were perfused, and microalgae reached all renal vasculature, without inducing damage. After perfusion and flushing, no signs of tissue damage were detected, and recovered microalgae survived the process. Altogether, this work proposes the use of photosynthetic microorganisms as vascular oxygen factories to generate and deliver oxygen in isolated organs, representing a novel and promising strategy for organ preservation.

## 1 Introduction

The lack of appropriate tissue oxygenation represents a major issue in several medical areas, being particularly relevant in the transplantation field, where organ ischemia induces hypoxia, limiting their *ex vivo* preservation time, as well as their further clinical outcome after transplantation (Hosgood et al., 2012; Giwa et al., 2017).

Aiming to decrease the oxygen requirements of isolated organs, static cold storage (SCS) has been the gold standard in clinical transplantation settings, reducing metabolism and therefore oxygen consumption in about 90% (Karangwa et al., 2016). However, this technique is limited in terms of preservation time and for maintaining the integrity of suboptimal grafts derived from expanded criteria donor (ECD) and donation after circulatory death (DCD), which are more sensitive to damage (Soo et al., 2020; Vries et al., 2020). SCS represents the first step in the cascade of ischemia reperfusion injury ensuing organ implantation (Eltzschig and Eckle, 2011), triggering tissue damage through oxidative stress and inflammation (Slegtenhorst et al., 2014). Moreover, it is associated with organ allograft dysfunction and acute rejection, reducing the graft survival (Zhao et al., 2018).

Aiming to provide oxygen *ex vivo*, novel technologies have been established. Among them, the use of machine perfusion is promising. Some of these systems are based on extracorporeal oxygenation devices, where erythrocytes are oxygenated in a dynamic system that allows them to recirculate through the organ vascular network. Although recent clinical data support their safety and efficacy (Xu et al., 2020; Tatum et al., 2021), the use of blood generates additional complications, because it is not always available on site, has a relative short preservation time and requires the inclusion of a membrane oxygenator, which substantially increases the total cost (Aburawi et al., 2019). Additionally, blood recirculation generates hemolysis, which can be toxic as free hemoglobin can cause inflammation and oxidative stress (Gkoumassi et al., 2012; Bodewes et al., 2021), thus the development of novel perfusable solutions for *ex vivo* oxygenation is an active field of research. The use of oxygen carriers as an alternative to erythrocytes has been widely studied, and promising results have been described for hemoglobin-based oxygen carriers obtained from annelids (e.g. *Arenicola marina*) (Bhattacharjee et al., 2020; Bodewes et al., 2021). However, they require intensive purification for their use, and their passive oxygen release kinetics makes them poorly controllable depending on the organ metabolic needs (Le Meur et al., 2020; Varney et al., 2021). As a purely synthetic alternative, perfluorocarbons have limitations due to the difficulty of controlling the kinetics of oxygen release, as well as the complexity of manufacturing and the need to incorporate them into different emulsions, limiting their widespread adoption in organ preservation (Malchesky, 2017; Jägers et al., 2021). As an alternative method for oxygen supply, we and others have proposed that the induction of local photosynthesis could modulate oxygen tension in hypoxic tissues. Based on this, photosynthetic therapies aim to generate a local symbiotic relationship between animal and photosynthetic cells where, in the presence of light, both metabolisms could be coupled with each other (Chávez et al., 2020). This approach has been described by our group in recent *in vitro* (Hopfner et al., 2014; Centeno-Cerdas et al., 2018; Chávez et al., 2021) and *in vivo* (Schenck et al., 2015; Chávez et al., 2016) studies, and further confirmed by other independent groups, highlighting its potential application in several medical fields, including tissue engineering and regeneration (Yamaoka et al., 2012; Evron et al., 2015; Haraguchi et al., 2017; Chen et al., 2020), heart ischemia (Cohen et al., 2017), and tumor treatment (Huo et al., 2020; Liu et al., 2020; Qiao et al., 2020). Moreover, an ongoing clinical trial is confirming its safety for tissue regeneration in humans (Obaíd et al. (2021) accepted for publication. ClinicalTrials.gov Identifier: NCT03960164).

Taking all this into consideration, in this work we propose that the development of perfusable photosynthetic solutions could allow organ preservation by *in situ* vascular oxygenation. To accomplish this long-term goal, here we describe the first generation of a perfusable photosynthetic solution for organ preservation, providing key scientific insights about its therapeutic potential.

## 2 Materials and Methods

### 2.1 Microalgae Culture

Cell-wall deficient UVM4-GFP *C. reinhardtii* strain (cw15-30-derived) was kindly provided by Prof. Jörg Nickelsen (LUM, Germany) and cultured as described before (Neupert et al., 2009). Briefly, microalgae were grown photomixotrophically at room temperature (20–25°C) on either solid Tris Acetate Phosphate (TAP) medium with 1.5% (w/v) agar or in bottles containing different volumes of liquid TAP medium placed in an orbital shaker (180 rpm). For light stimulation, a lamp with the full spectrum of white light was used to provide continuous light exposure (30 μE/m^2^ s) (Harris, 2009). Cell concentration was determined using a Neubauer chamber.

### 2.2 Generation of the Photosynthetic Solution for Organ Preservation

To prepare the PSOP, Ringer’s lactate solution was mixed with 0.5% (w/v) mannitol (RLM; AppliChem Panreac) as impermeant agent (Nicholson and Hosgood, 2017). Liquid cultures of *C. reinhardtii* in exponential growth phase were grown in 100 ml borosilicate glass bottles up to 5 L depending on the requirements of each experiment. For optimal growth, constant agitation was maintained, either on an orbital shaker or a magnetic stirrer. For harvesting, liquid cultures up to 2 L were centrifuged and 5 L cultures were recovered by decantation; thereafter pelleted algae were resuspended in RLM at different cell densities (10^6^–10^9^ C. reinthardtii/ml). As control groups, TAP medium and a mixed solution of TAP:RLM (in 1:1 ratio) were included. For *ex vivo* kidney perfusion (see below in section 2.10 and section 2.12) and metabolic coupling assay of kidney slices (section 2.8), 5% (w/v) dextran-70 (H979; AK Scientific Inc) was added to RLM to maintain the oncotic pressure.

### 2.3 Microalgae Viability Assays

After 24 h of incubation of *C. reinhardtii* in RLM, TAP or TAP:RLM, viability of the microalgae was determined by examining growth after 5 days of inoculation in agar plates. As viability probe (Jamers et al., 2009), microalgae were diluted to 3 × 10^5^ *C. reinhardtii*/ml and incubated for 1 h with 25 µM of Fluorescein diacetate (FDA, F1303, Life Technologies). A death control was included by heating *C. reinhardtii* at 85°C for 10 min and 10^5^ events per sample were acquired in BD Influx cell sorter (Becton Dickinson). Data was analyzed with FlowJo software (Becton Dickinson) by gating chlorophyll positive cells. In order to discard the contribution of GFP to the FDA signal, unstained, stained, and dead microalgae were used as controls to determine the basal level of fluorescence and set the gate for FDA.

### 2.4 Cell Morphology Evaluation

General morphology of the microalgae in RLM, TAP or TAP:RLM was evaluated by optical microscopy (Leica DM500), as well as by flow cytometry (BD FACSCanto II analyzer, Becton Dickinson). Cell diameter was quantified using 4, 6, 10 and 15 µm size marker beads (F13838; Life Technologies), and 10^5^ events were recorded in the microalgae gate. Data was analyzed with FlowJo software (Becton Dickinson) by gating chlorophyll positive cells.

### 2.5 Oxygen Production of PSOP

After 0 and 24 h of incubation in RLM, the oxygen production of PSOP containing different cell densities (0, 10^6^, 10^7^, 10^8^ and 10^9^ *C. reinhardtii*/ml) was measured at 28°C using an Oxygraph System (Hansatech Instruments). Samples (1 ml) were subjected to 10 min of darkness, followed by 10 min of red (455 nm) and blue (630 nm) illumination (8.7 μE/m^2^ s). Oxygen production rate was calculated from the slope of oxygen evolution. Data was normalized and expressed as the oxygen produced by each microalga cell per second.

### 2.6 PSOP Osmolarity and Viscosity

The osmolality of PSOP containing different cell densities (0, 10^6^, 10^7^, 10^8^ and 10^9^ *C. reinhardtii*/ml) was measured at RT using a cryoscopic osmometer (Osmomat 030, Gonotec). Viscosity was measured in a rheometer, using a 40 mm conical geometry in response to different shear rates (Discovery HR-2, TA Instruments). The gap between the sample and the geometry was set at 300 µm and measurements were carried out at 28°C.

### 2.7 *In vivo* Toxicity Assay

Zebrafish is a highly characterized and validated model for biomedical toxicity assays (Choi et al., 2021), thus ten larvae at 5 days post fertilization (*Danio rerio,* TAB5 strain) were obtained from our breeding colony and incubated at 28°C, in 12-well plates, and exposed for 24 h to medium (E3, control) or PSOP with increasing cell densities of *C. reinhardtii*. In order to maintain larvae in optimal conditions, incubations were performed in a 14:10 light-dark photoperiod as we described before (Alvarez et al., 2018), and no additional illumination was provided to induce photosynthetic oxygen production. Then, survival was determined as the percentage of heart beating larvae. For morphological imaging, larvae were anesthetized by immersion in 4.2% (w/v) tricaine (Sigma-Aldrich), euthanized by cold shock (−20°C for 10 min), fixed in 4% paraformaldehyde and imaged with a stereoscope (Leica S6D).

### 2.8 Metabolic Coupling Assay With Larvae and Rat Kidney Slices

Twenty zebrafish larvae (5 dpf) contained in 1 ml of RLM were added into the electrode chamber of the Oxygraph System (Hansatech Instruments), and the oxygen evolution was measured for 5 min in darkness followed by 5 min of red (455 nm) and blue (630 nm) illumination (8,7 μE/m^2^ s). Then, 200 µL of PSOP containing 10^8^ *C. reinhardtii*/ml were added (10^6^ *C. reinhardtii*/larva), and the oxygen evolution was recorded for the next 10 min in the same lighting condition, followed by 10 min of darkness. Oxygen metabolic rate was calculated from the linear slope of oxygen concentration curve. For rat kidney slices the same setting was applied with slight modifications. Male Sprague-Dawley rats (250–400 g) were obtained from the animal facility of INTA, Universidad de Chile (Santiago, Chile). All animal experiments were performed according to protocols approved by the Ethics Committee of Pontificia Universidad Católica de Chile (180813015). Animals were anesthetized with ketamine (90 mg/kg)/xylazine (10 mg/kg) i. p. and the kidneys were washed out of blood by perfusing 5 ml warm RLM solution supplemented with 5% (v/w) dextran-70 through the abdominal aorta with a syringe. The left kidney was excised, cut in half, and mounted in a vibratome to obtain coronal slices. A single central slice (500 µm thick) was incorporated in the oxygraph chamber and incubated with 2 ml of dextran supplemented RLM, and 100 µL containing 2·10^7^ *C. reinhardtii* were added.

### 2.9 Porcine Kidneys Procurement

Female healthy pigs were selected by weight (35–45 Kg) from our research breeder facility (CICAP-UC Pirque, Santiago, Chile). Animals were sedated with ketamine (25 mg/kg) and midazolam (0.5 mg/kg) and general anesthesia was maintained with 2% isoflurane and animals were connected to mechanical ventilation. Heparin (100–200 UI/kg) was administered to avoid coagulation during organs procurement. Kidneys were isolated and perfused with 500 ml of Custodiol^®^ and kept at 4°C until experimental studies. Thereafter, pigs were euthanized with thiopental and potassium chloride. All the experiments were performed after the approval of our local ethical committees (approval No. 160126009).

### 2.10 Microalgae Distribution After *ex vivo* Kidney Perfusion

Porcine kidneys were manually perfused with 50 ml of PSOP (5 × 10^8^ *C. reinhardtii*/ml) and submitted to macroscopic and histological analysis. For low magnification imaging, fresh organs were sliced with a surgical scalpel and pictures were taken using a stereoscope (Leica S6D). Then, biopsies were fixed in 4% paraformaldehyde, included in O.C.T. compound (4,583, Sakura), sliced (30 µm), stained with H&E and imaged with Leica DM500 microscope.

### 2.11 Machine for Dynamic Organ Perfusion

A machine perfusion system was specially designed and manufactured for this study (Sky-Walkers SpA), which consisted of an organ receiving chamber, a volume reservoir, and a centrifugal pump (EC042B IDEA^®^ Motor, Pittman) connected to a reservoir, to carry out the flow to the renal artery. Pressure (TruWave disposable pressure transducers, Edwards Lifesciences) and flow sensors (Biomedicus TX50 Bio-probe flow transducer, Medtronics) were placed in the arterial line and a closed-loop pressure control was designed and manufactured to keep infusion pressure stable within physiological ranges (70–80 mmHg).

### 2.12 Dynamic Sub-normothermic *ex vivo* Perfusion of Porcine Kidneys

Isolated kidneys were cannulated through aortal patch and connected to the perfusion machine described in section 2.11. Kidneys were perfused at RT for 30 min with continuous recirculating flow, using 1 L of PSOP at 5 × 10^7^ *C. reinhardtii*/ml. Then, organs were flushed with RLM containing 5% dextran-70 (w/v) for 40 min, without recirculation. Mean arterial pressure (MAP) and perfusion flow were recorded, and renal vascular resistance (RVR) was calculated as: RVR = MAP/perfusion flow. Samples of PSOP before and during perfusion/flushing were diluted to 3 × 10^5^ *C. reinhardtii*/ml, incubated with FDA and analyzed by flow cytometry as described in section 2.3. After perfusion and flushing, biopsies were fixed in 4% paraformaldehyde, included in paraffin, sliced (4 µm) and stained with H&E.

### 2.13 Statistical Analysis

All assays were performed in at least three independent experiments (unless specified). GraphPad Prism five software (GraphPad Software) was used for statistical analyses. Statistical tests used are described in each result section.

## 3 Results

### 3.1 Functional Characterization of PSOP

**FIGURE 1:**
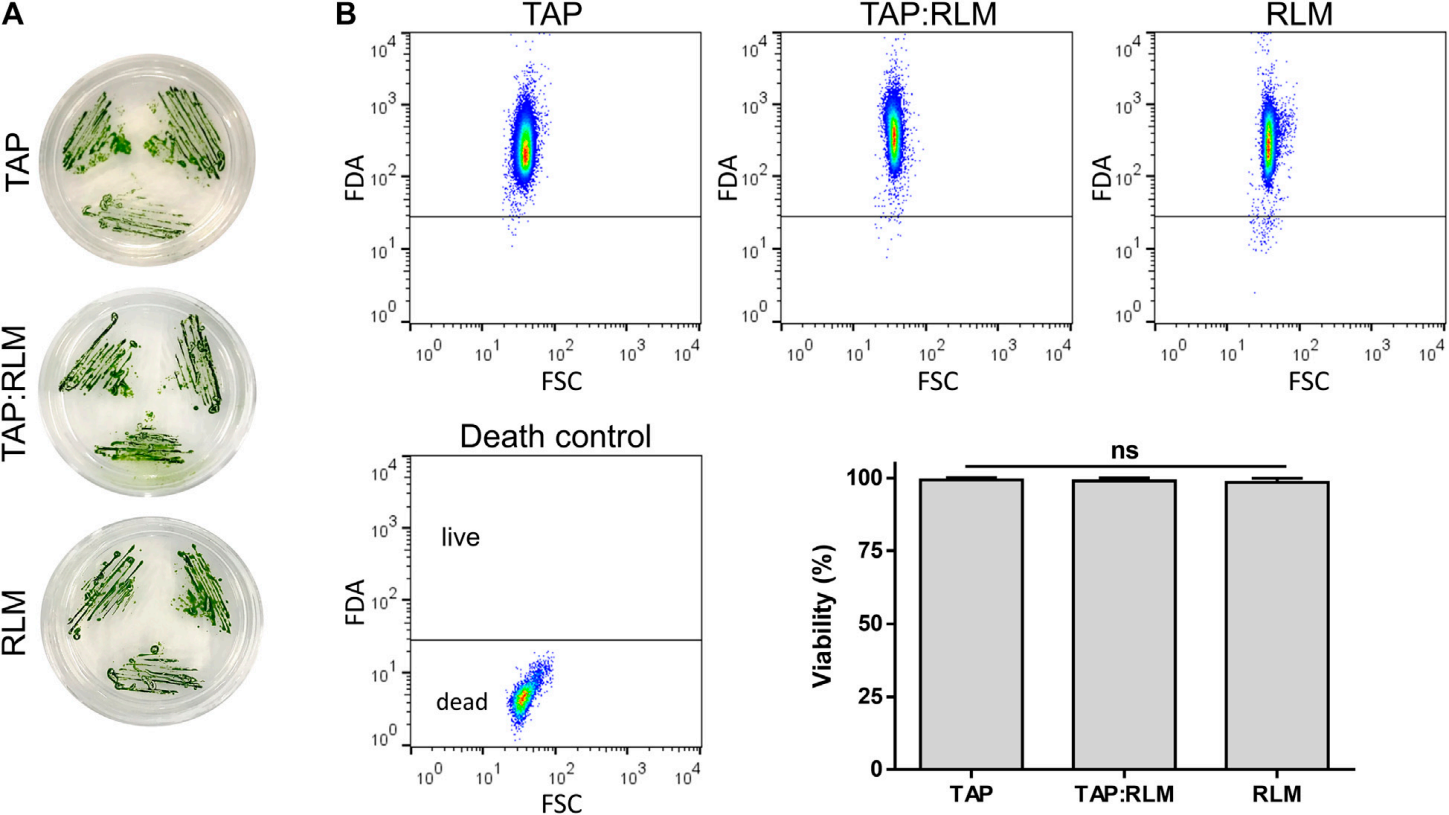
Viability of *C. reinhardtii* in RLM. *C. reinhardtii* were incubated for 24 h in their culture media (TAP), a standard solution for organ preservation (RLM) or a mix of both in a 1:1 ratio (TAP:RLM). Viability of microalgae was not affected as shown by their growing capacity **(A)** and by flow cytometry **(B)**. Data are expressed as mean ± SD; N = 3; ns: non-significant (one-way ANOVA test).

**FIGURE 2:**
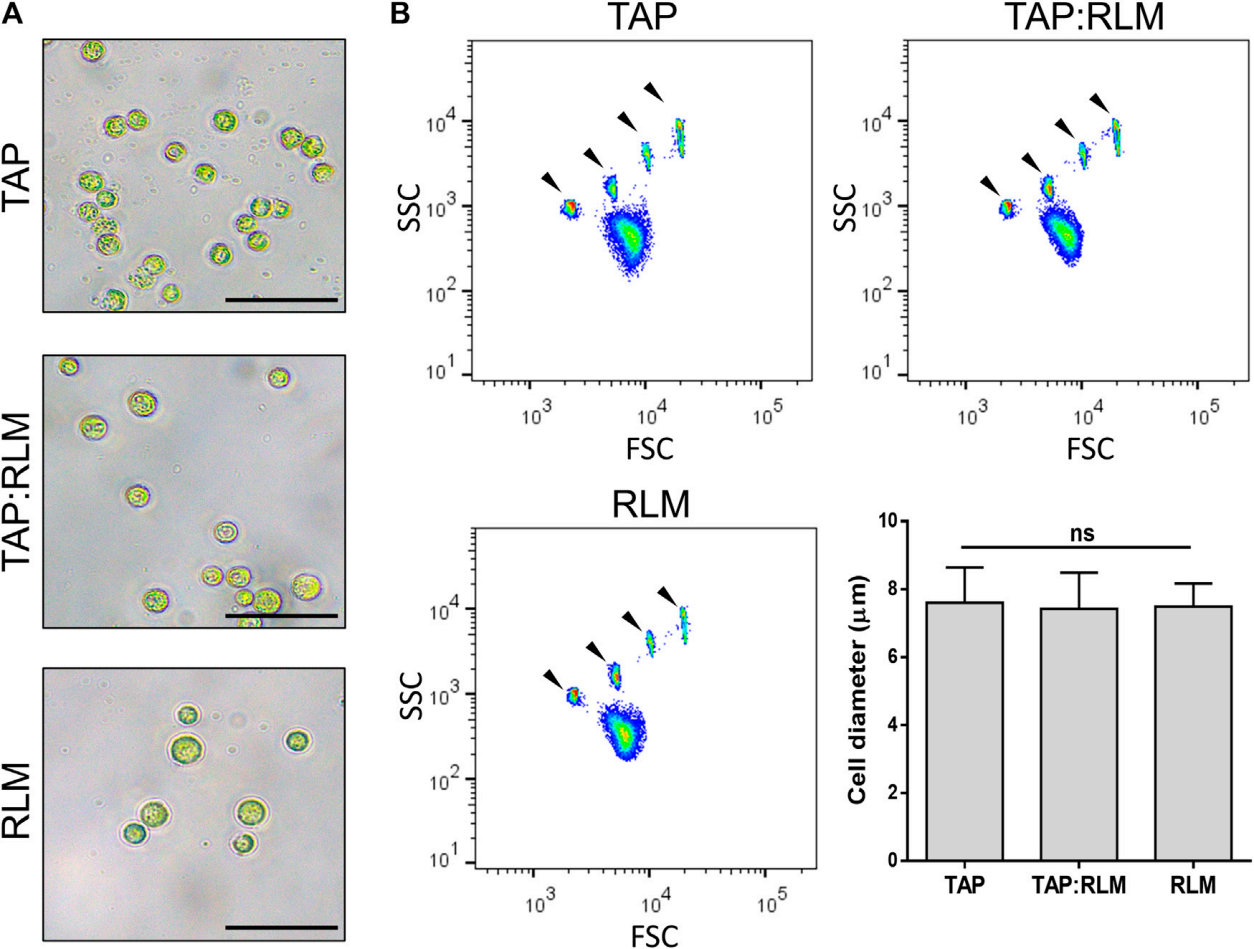
Morphology of *C. reinhardtii* in RLM. *C. reinhardtii* were incubated for 24 h in their culture media (TAP), a standard solution for organ preservation (RLM) or a mix of both in a 1:1 ratio (TAP:RLM). Morphology **(A)** and size **(B)** of the microalgae were not affected by the media. Arrow heads indicate size marker beads of 4, 6, 10, and 15 µm in diameter, from left to right. Scale bars represent 25 µm. Data are expressed as mean ± SD; N = 3; ns: non-significant (one-way ANOVA test).

**FIGURE 3:**
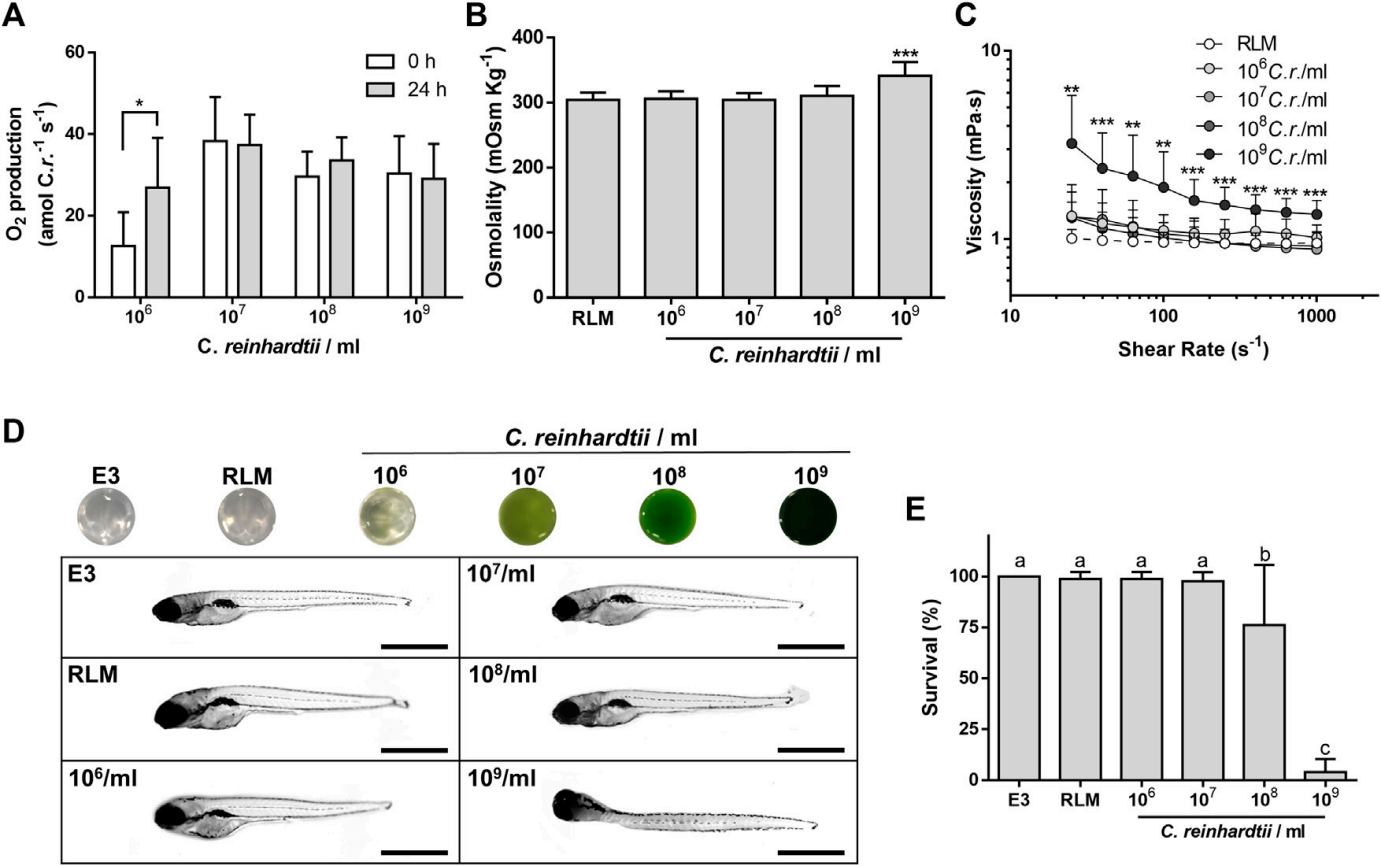
Characterization of the photosynthetic solution. Different densities of *C. reinhardtii* were added to a standard solution for organ preservation (RLM). The oxygen production rate of microalgae was maintained after 24 h, except for 10^6^ *C. reinhardtii*/ml, which increased **(A)**. Up to 10^8^ *C. reinhardtii*/ml, no significant differences were observed in the osmolality **(B)** and rheological properties of the solution **(C)**. Zebrafish larvae were exposed for 24 h to photosynthetic solution containing different densities of microalgae, in the dark. Up to 10^8^ *C. reinhardtii*/ml larvae presented normal phenotypes compared to control [E3; **(D)**]. Mild and severe mortality were observed at 10^8^ and 10^9^ *C. reinhardtii*/ml, respectively **(E)**. Scale bars represent 1 mm in D. Data are expressed as mean ± SD; N = 3, 4; *p < 0.05, ***p ≤ 0.001 (one-way ANOVA followed by Tukey’s test in A; one-way ANOVA followed by Dunnett’s test in B; two-way ANOVA followed by Sidak´s test in C); different letters in E indicate significant differences with *p* < 0.05 (one-way ANOVA followed by Tukey´s test).

The first step to study the feasibility of using photosynthetic microalgae in a perfusable solution for *ex vivo* organ preservation was to evaluate whether the microalgae *C. reinhardtii* could survive in a standard perfusable physiological solution for organ preservation. Thus, microalgae were incubated for 24 h in RLM, standard algae medium (TAP) or a 1:1 mixture of both. Then, cell viability was assessed, both by evaluating their growing capacity in agar plates (Figure 1A) and by cell cytometry (Figure 1B). No significant differences were observed among groups, confirming that *C. reinhardtii* remain viable for at least 24 h in RLM. Then, possible morphological changes of *C. reinhardtii* induced by incubation in the RLM were also evaluated. As shown in Figure 2A, the general morphology and size of the microalgae did not vary, being further quantified by flow cytometry, where cell diameters of 7.5 ± 0.3, 7.4 ± 0.5 and 7.6 ± 0.4 µm were observed for RLM, TAP:RLM and RLM respectively (Figure 2B). Afterwards, oxygen production rates of RLM containing different densities of *C. reinhardtii* (PSOP) were characterized and compared. No significant differences in oxygen production were observed between 0 and 24 h of incubation in RLM, nor among the different densities, except for the group containing 10^6^ *C. reinhardtii*/ml, which presented a higher production rate after 24 h of incubation (Figure 3A). Within the studied range, results show that single cell oxygen production was conserved at higher densities, remaining in the 30–40 (amol/cell s) range. Interestingly, no significant differences in cell number were observed after 24 of incubation, suggesting a decrease in the proliferation capacity of the microalgae in this particular experimental setting. Because maintaining osmotic pressure under physiological ranges is a fundamental requirement for perfusable solutions for organ preservation, osmolality was also quantified. As shown in Figure 3B, the presence of microalgae did not affect the osmolality of the PSOP, remaining in the range of 310 mOsm/Kg, except for 10^9^ *C. reinhardtii*/ml where the osmolality increased significantly. The effect of the microalgae density in the rheological properties of the PSOP was also studied, measuring viscosity in response to increasing shear rates. Once again, up to densities of 10^8^ *C. reinhardtii*/ml, viscosity values were comparable to RLM, while at 10^9^ *C. reinhardtii*/ml, the solution presented significantly higher viscosity (Figure 3C).

To evaluate potential toxic effects of the PSOP, zebrafish larvae were used as an *in vivo* vertebrate model for toxicity testing. After incubation, no obvious morphological changes, or signs of damage (such as edema formation and eye size reduction) were observed at different microalgae densities, except for the 10^9^ *C. reinhardtii*/ml, which induced a general curvature and twisting of the larvae (Figure 3D). In terms of viability, solutions containing up to 10^7^ *C. reinhardtii*/ml were non toxic for the larvae, while densities of 10^8^ and 10^9^ *C. reinhardtii*/ml, induced mild and severe mortality respectively (Figure 3E).

### 3.2 Metabolic Coupling Between PSOP and Animal Systems

**FIGURE 4:**
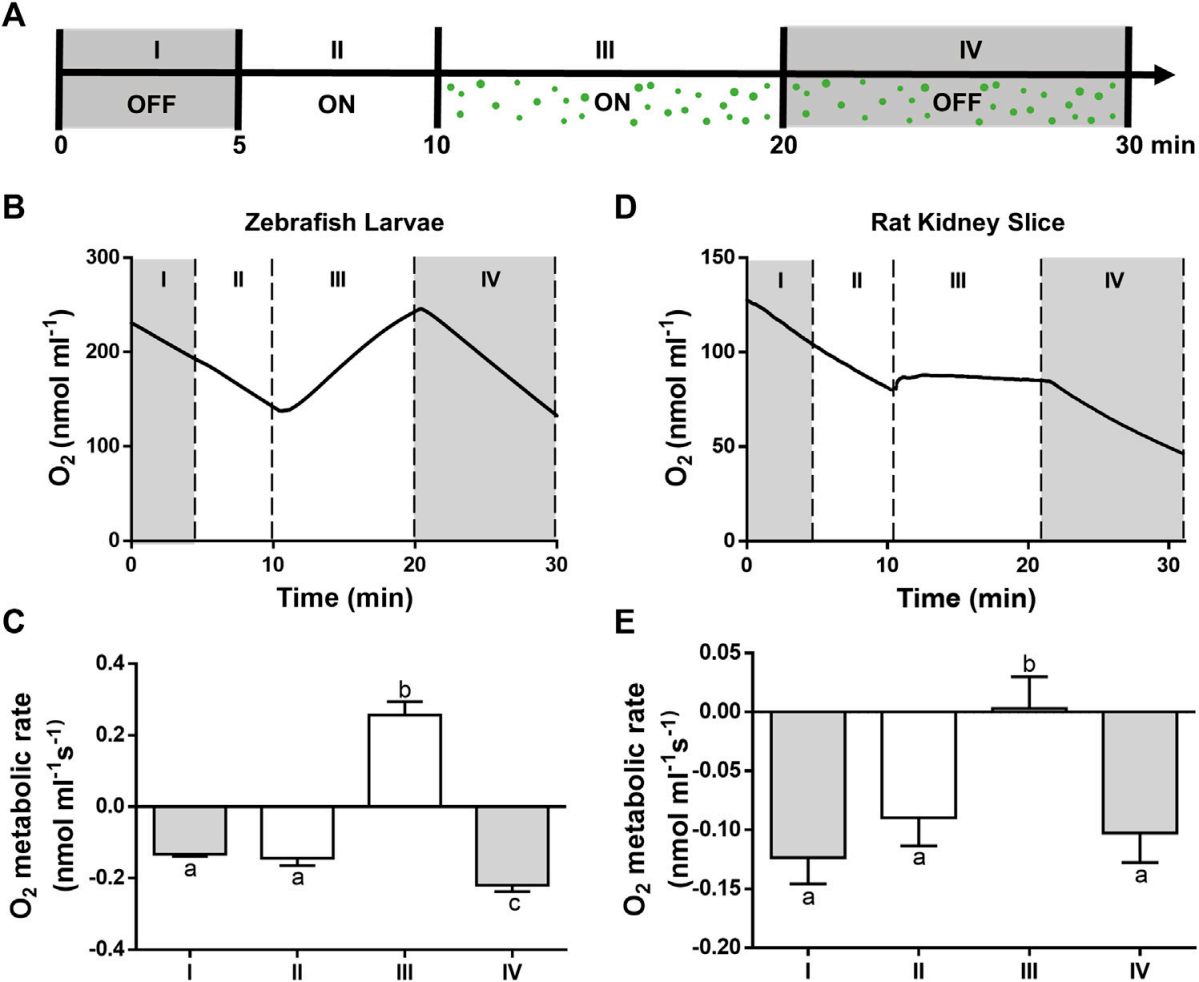
Oxygenation capacity of the photosynthetic solution. Oxygen concentrations were measured for 5 min in darkness (I, OFF) or light (II, ON). Then, microalgae were incorporated and measurements were performed for 10 min in the presence (III, ON) or absence (IV, OFF) of light **(A)**. In the absence of light or microalgae (I, II and IV) a negative slope of the curve was seen for both, zebrafish larvae **(B,C)** and kidney slices **(D,E)**, while in the presence of light and microalgae, the slope became positive for the larvae and nearly flat for the slices. A representative curve is shown for each experiment **(B–D)** and their metabolic rates calculated from the slopes **(C–E)**. Data are expressed as mean ± SD; N = 3; different letters in C and E indicate significant differences with *p* < 0.05 (one-way ANOVA followed by Tukey´s test).

The capacity of PSOP to produce enough oxygen to support the metabolic requirements of an active biological system was evaluated. First, zebrafish larvae were placed in an Oxygraph chamber containing RLM, and the oxygen evolution was measured in the absence or presence of microalgae, as well as in the absence or presence of light (Figure 4A). In the absence of microalgae, the oxygen concentration decreased overtime, and the negative slope of the curve did not vary in the presence of light, indicating high oxygen consumption of larvae at this stage (Figures 4B,C, segment I and II). Afterwards, microalgae were added to the chamber, and the slope of the curve immediately showed a positive slope, meaning that the oxygen production of PSOP exceeded the consumption rate of the larvae (Figures 4B,C, segment III). Finally, the light was turned off and a negative slope was observed again (Figures 4B,C, segment IV). To validate this data with a more relevant model for transplantation, the same setting was applied to fresh rat kidney slices, and similar results were obtained, as in the presence of light and microalgae, the negative slope of the curve was reverted in segment III, becoming nearly flat, indicating equal oxygen production and consumption; and finally turning negative again in segment IV upon turning off the light (Figures 4D,E).

### 3.3 Characterization of *ex vivo* Perfused Porcine Kidneys

**FIGURE 5:**
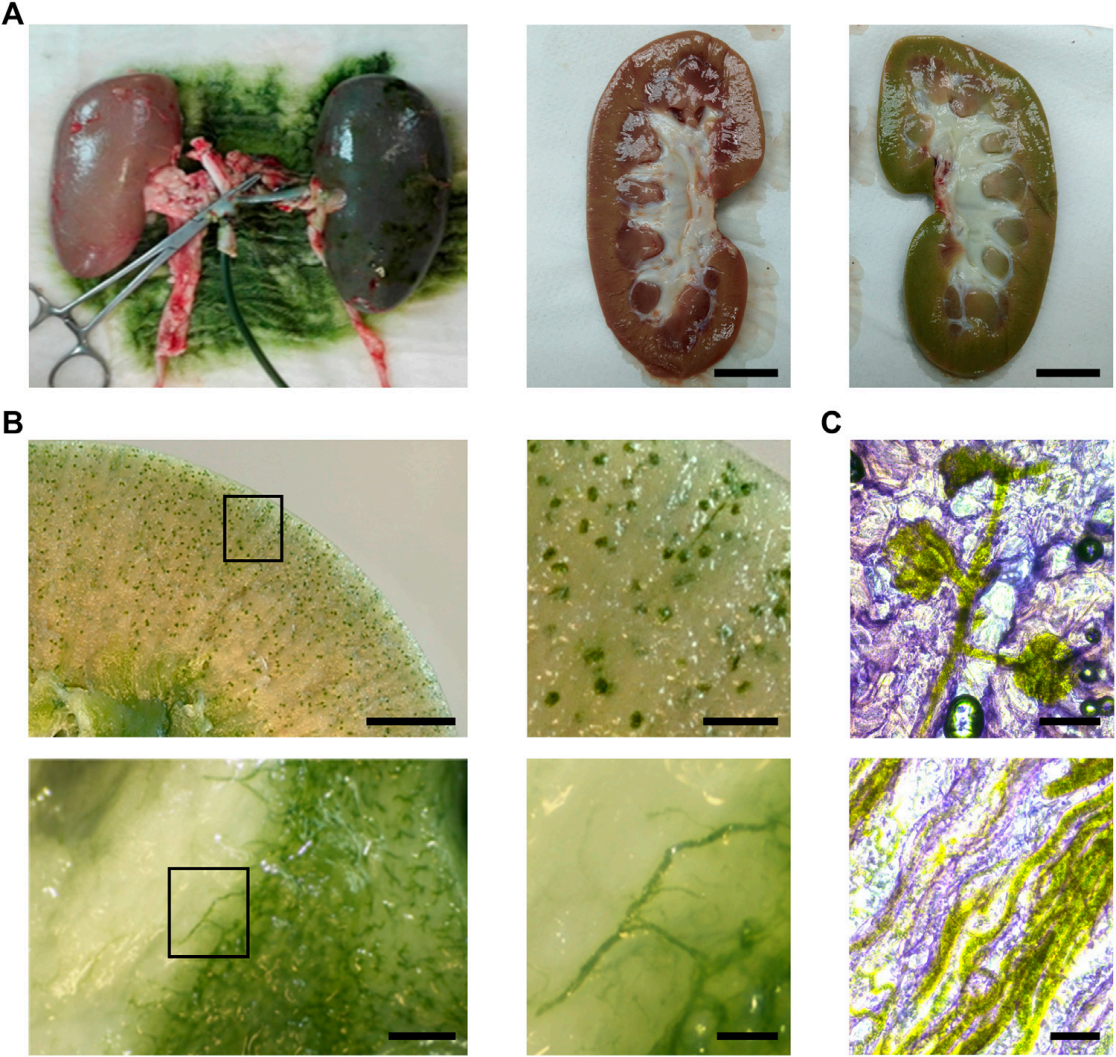
Distribution of the photosynthetic solution in porcine kidney. Compared to controls, organs turned green after perfusion [**(A)**, left and right]. Fresh slices show a vascular distribution of the solution in the renal cortex [**(B)**, top] and medulla [**(B)**, bottom]. Cryosections of perfused kidneys show the distribution of *C. reinhardtii* in glomeruli and afferent arteriole [**(C)**, top] and medullar blood vessels and capillaries [**(C)**, bottom]. Scale bar represents 2 cm in A, 5 mm in **(B)** (top, left), 1 mm in **(B)** (top, right) and **(B)** (bottom, left), 250 µm in **(B)** (bottom, right), 100 µm in **(C)**.

In order to validate the PSOP in a clinically relevant model, isolated porcine kidneys were manually perfused, and renal tissue turned uniformly green, presenting an apparent slight swelling. Besides, no other macroscopic differences were observed among the non-perfused and perfused organs (Figure 5A). Then, fresh perfused tissues were sliced and observed under a stereoscope, clearly showing that microalgae reached the entire vascular territory of the organs. In contrast to calyxes and papilla, where the vascular density is lower, the cortex as well as the medullar pyramid exhibited an intense green color (Figure 5B). A more detailed localization of the microalgae was microscopically evaluated in cryosections, which showed their homogeneous presence through the entire vascular structures, including the globular distribution in the glomeruli with the afferent arteriole and the characteristic parallel line arrangement of the vessels in the renal medulla (Figure 5C).

**FIGURE 6:**
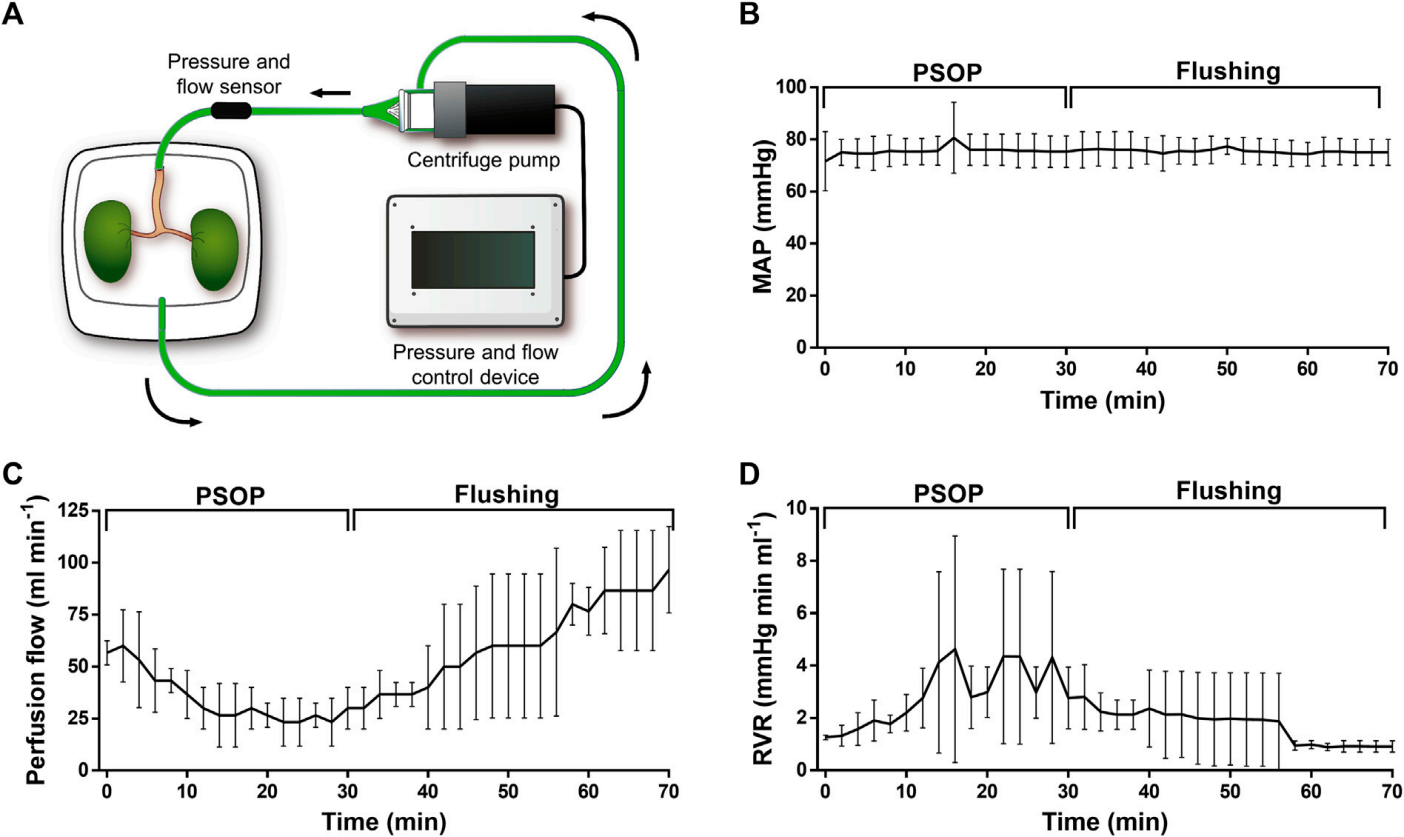
Dynamic perfusion of isolated porcine kidneys. Schematic representation of the *ex vivo* perfusion system, containing a pressure-flow controlling device, a centrifuge pump, and a container for the isolated organs **(A)**. Vascular parameters were measured during the photosynthetic perfusion and the subsequent flushing step. Mean arterial pressure (MAP) was set to 70–80 mmHg remaining stable during the entire procedure **(B)**. Perfusion flow decreased during the PSOP perfusion **(C)**, while renal vascular resistance (RVR) increased, recovering during the flushing step **(D)**. Data are expressed as mean ± SEM; N = 3 in **(B–D)**.

**FIGURE 7:**
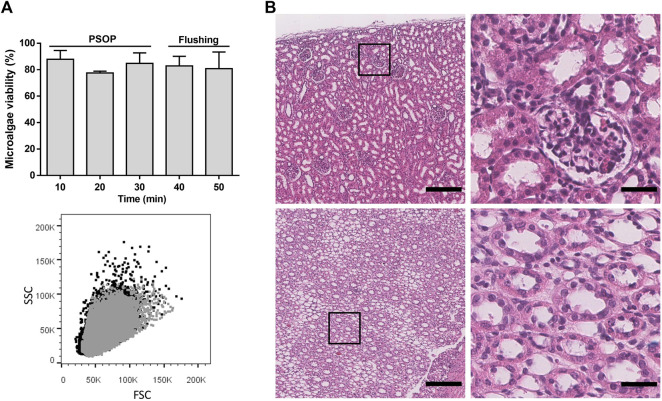
Microalgae viability and renal tissue integrity after dynamic photosynthetic perfusion. Viability [**(A)**, upper] and morphology [**(A)**, lower] of the microalgae were not affected by the perfusion and the subsequent flushing step. H&E-stained paraffin sections, shows a normal histological structure of porcine kidneys in cortex [**(B)**; top] and medulla [**(B)**; bottom] after perfusion. Black and grey dots in A (bottom) indicate microalgae samples of the solution obtained before and after 10 min of perfusion, respectively, showing an almost complete overlapping of the signal where gray dots masked the microalgae population represented by black dots. Scale bar represents 200 µm [**(B)**, left] and 30 µm [**(B)**, right]. Data are expressed as mean ± SD; N = 2 in A.

To continue the PSOP validation, perfusion dynamics were evaluated in isolated porcine kidneys, which were connected to an organ perfusion machine prototype specially designed for this study (Figure 6A). This device has automatic flow control based on the sensed values to maintain physiological pressure throughout the procedure. The results showed a stable MAP of 75.5 mmHg during perfusion and the following flushing step, confirming the reliability of the perfusion machine (Figure 6B). After the first 5 min, the flow decreased from a mean of 56.7 ± 3.3 to 26.7 ± 8.8 ml/min by 15 min, remaining constant until the flushing step with microalgae-free solution, where the flow gradually recovered and reached values up to 96.7 ± 12.0 ml/min (Figure 6C). Accordingly, during PSOP perfusion, calculated RVR increased from 1.26 ± 0.05 to a peak of 4.63 ± 2.49 mmHg min/ml at 16 min, being then less stable with variations between 2.79 ± 0.69 and 4.31 ± 1.89 mmHg min/ml. Thereafter, the initial RVR values were restored after 28 min of flushing (Figure 6D). Next, the effect of the perfusion itself in the integrity of the microalgae was studied. Here, samples were collected from the renal vein effluent and analyzed by flow cytometry. Interestingly, the perfusion process did not affect microalgae viability, remaining at about 80% during the perfusion (83.4 ± 6.7%) and the flushing step (81.8 ± 8.5%) (Figure 7A, upper). In addition to viability, the effect of perfusion in the morphology of the microalgae was also assessed, finding an almost complete overlapping between the microalgae populations obtained before and after perfusion (Figure 7A, lower). Finally, tissue integrity was analyzed after perfusion and flushing, and H&E-stained paraffin sections showed the classic kidney architecture, represented by the cortex, containing renal corpuscles, proximal and distal tubules, and the medulla with collecting ducts and loops of Henle (Figure 7B). Overall, results show that glomeruli and tubules did not present signs of damage, as neither necrotic cells in Bowman’s capsule nor blood cells or microalgae in Bowman’s space were detected.

## 4 Discussion

In this proof-of-concept study, we demonstrate the feasibility to incorporate photosynthetic microorganisms in organ perfusion solutions to provide an alternative intravascular source of oxygen to isolated organs. This concept is based on our previous research in implantable photosynthetic biomaterials (Hopfner et al., 2014; Schenck et al., 2015; Chávez et al., 2016; Chávez et al., 2021; Centeno-Cerdas et al., 2018), and could potentially generate a new physiological state of *normoxic ischemia*, where the lack of blood supply may not necessarily trigger hypoxia. The use of photosynthetic microorganisms as local oxygen factories may have significant advantages compared to the standard approaches. Among others, there is no need for additional carriers, and the local oxygen release kinetics could be easily controlled by the light intensity provided (Rühle et al., 2008). Additionally, this approach allows the generation of genetically modified photosynthetic organisms that, in addition to oxygen, could locally release fresh bioactive recombinant molecules as well (Chávez et al., 2020).

Ringer’s lactate solution was chosen as the base to develop the PSOP because of its extended clinical use as physiological fluid (Singh and Davis, 2018; de Sousa et al., 2021). Mannitol was added as an impermeant agent to prevent cell swelling, one of the main requirements for preservation solutions (Nicholson and Hosgood, 2017). For kidney perfusion and oxygraphy of kidney slices, dextran-70 was also added to maintain the oncotic pressure and prevent tissue edema (Wang et al., 2004; Wilson et al., 2006). Although cell proliferation was not observed after 24 h of incubation in either medium (RLM and TAP), Ringer’s lactate and mannitol solution (RLM) showed high biocompatibility with *C. reinhardtii*, without affecting microalgae viability and morphology, nor their photosynthetic capacity overtime. This result is somehow surprising because preservation solutions have to meet specific physicochemical requirements (Fuller et al., 2018) that differ from the optimal culture conditions of the microalgae. For instance, the osmolality of the microalgae medium (TAP) is around 64 mOsm/Kg (Komsic-Buchmann et al., 2012) while it is 305 mOsm/Kg for RLM, and previous studies have shown how an increase in osmotic stress can negatively affect cell growth and photosynthetic rates in *C. reinhardtii* (Neale and Melis, 1989). With exception of the highest microalgae density, all tested PSOP presented the same flowing behavior and dynamic viscosity values as RLM. It is worth noting that in all groups viscosity tended to decrease with higher shear rates (shear thinning), resembling blood behavior (Cherry and Eaton, 2013) and exhibiting appropriate rheological properties for organ perfusion. Moreover, at most concentrations of microalgae, the photosynthetic solution was biocompatible with zebrafish larvae. This toxicity model was chosen because is extensively used in several fields of research (Lieschke and Currie, 2007), and has been widely validated as a reliable model to test toxicity for biomedical application (Choi et al., 2021). In fact, the sensibility of this model shows that both, morphology and viability were significantly affected by incubations in PSOP at the highest density of microalgae, allowing to better define the potential clinical range for a safe microalgae perfusion procedure. This toxic effects at high microalgae densities could be due to several reasons including a high oxygen consumption of the microalgae under such insufficient illumination conditions, or due to issues related to the increased viscosity and osmolality of the PSOP.

To evaluate the functionality of PSOP in a more clinical setting it is required to provide adequate illumination. Hence, an important limitation of this study is that, due to the lack of established technologies for optimal inner organ illumination, here we could not evaluate the functional effect of the PSOP in the oxygenation and further preservation of isolated organs, supporting the need of developing accurate illumination devices for this purpose. Nevertheless, we decided to evaluate the oxygenation capacity of PSOP in zebrafish larvae. In addition to the advantages described above, in contrast to other organisms, larvae are fully permeable, thus their entire gas interchange occurs by diffusion, allowing to quantify the metabolic interaction between the photosynthetic oxygen produced by the PSOP and the living animal tissues. Considering that five dpf larvae mass are approximately 0.5 mg each (Barrionuevo et al., 2010), it follows that 10^9^ microalgae suspended in RLM would be sufficient to oxygenate 1 G of tissue. However, because larval stages are highly hypermetabolic, this number of microalgae might be overestimated. Oxygen requirements of human cells can widely vary ranging from values below 1 to 350 amol/cell s (Wagner et al., 2011). For example, human liver cells in culture are described to consume around 100 amol/cell s (Wagner et al., 2011), which is promising when compared to our results showing that each microalga in the PSOP solution is capable to produce 30–40 amol/cell s upon light exposure. Therefore, a ratio of 3:1 microalgae to cell would be sufficient to ensure optimal tissue oxygenation for that cell type, especially relevant when considering that hepatocytes are roughly 500 times larger in volume (Marguerat and Bähler, 2012) and the metabolic oxygen requirements of tissues decrease by 50% at sub-normothermic conditions (Karangwa et al., 2016). In fact, our results shows that 2×10^6^ microalgae were sufficient to match the oxygen consumption of a 500 µm-thick rat kidney slice, weighting approximately 45 mg. Interestingly, similar results have been recently published, showing that a suspension of *C. reinhardtii* was also capable to oxygenate mouse brain slices *in vitro* (Voss et al., 2021). The setting described above strongly resembles the famous experiment performed by Joseph Priestley in 1772 where he showed that, when placed in a close compartment, a plant can provide enough oxygen to supply the metabolic requirements of a mouse (West, 2014). However, before the clinical translation of this approach, several issues need to be addressed especially in terms of the evaluation of safety and efficacy of this concept in human organs. Additionally, the composition of the PSOP described here will need to be optimized according to each particular clinical application, including its chemical composition, photosynthetic strain, and illumination setting.

As kidney represent the most frequent organ transplanted worldwide (WHO, 2020), we decided to initially evaluate the perfusion dynamic of PSOP in isolated porcine kidneys. Additionally, due to the kidney’s intrinsic complexity, this approach allows to test the solution under highly challenging conditions, providing important information about its *ex vivo* rheological properties, and the effect of this perfusable solution in the different renal vascular domains. Interestingly, *C. reinhardtii* could reach even the smallest capillaries of porcine kidneys and survive the circulation process without damaging the general tissue architecture of the organs. However, a transient increase in the vascular resistance to flow was observed during 5×10^7^ cell/ml PSOP perfusion, which can be due mostly to a simple increase in fluid viscosity as inferred from the rheological characterization (Figure 3C). This analysis, together with the recovery of resistance upon flushing, minimized the probability that reversible vascular occlusion could occur in this setting.

To optimize the approach presented here, further research should explore the immense biodiversity of photosynthetic microorganisms to find the most appropriate cell that may fit to every different organ preservation setting. This is valid for cell size, and other key features such as optimal temperature, osmolality, oxygen production, or illumination requirements. For instance, photosynthetic microorganisms range widely in size, from *Ostreococcus tauri* with less than 1 µm of diameter, being the smallest free-living eukaryote known; and some other species such as *Entemoneis kufferati* and *Synechococcus lividus* that live in environments from 0 up to 72 Celsius degrees, respectively (Varshney et al., 2015).

As conclusion, this work provides the first evidence to support the use of photosynthetic perfusable solutions for organ preservation, representing another important step towards human photosynthesis and its potential therapeutic applications.

## Data Availability

All data associated with this study are in the paper, and can be shared with approved outside collaborators under a materials transfer agreement; requests should be sent to JTE jte@uc.cl or RR, rareboll@uc.cl.
